# Prognostic Role of Serum Cytokeratin-19 Fragment (CYFRA 21-1) in Patients with Hepatocellular Carcinoma

**DOI:** 10.3390/cancers12102776

**Published:** 2020-09-28

**Authors:** Gian Paolo Caviglia, Michela Ciruolo, Antonella Olivero, Patrizia Carucci, Emanuela Rolle, Chiara Rosso, Maria Lorena Abate, Alessandra Risso, Davide Giuseppe Ribaldone, Francesco Tandoi, Giorgio Maria Saracco, Elisabetta Bugianesi, Silvia Gaia

**Affiliations:** 1 Department of Medical Sciences, University of Turin, 10100 Turin, Italy; antonella.olivero@unito.it (A.O.); chiara.rosso@unito.it (C.R.); marialorena.abate@unito.it (M.L.A.); davrib_1998@yahoo.com (D.G.R.); giorgiomaria.saracco@unito.it (G.M.S.); elisabetta.bugianesi@unito.it (E.B.); 2 Division of Gastroenterology, Città della Salute e della Scienza University-Hospital, 10100 Turin, Italy; michela.ciruolo89@gmail.com (M.C.); pcarucci@cittadellasalute.to.it (P.C.); emanurolle@inwind.it (E.R.); alessandrarisso82@gmail.com (A.R.); 3 Liver Transplant Unit, General Surgery 2U, Department of Surgical Sciences, Città della Salute e della Scienza University-Hospital, 10100 Turin, Italy; francesco.tandoi@gmail.com

**Keywords:** AFP, BCLC, biomarker, HCC, PIVKA-II, prognosis

## Abstract

**Simple Summary:**

The prognosis of hepatocellular carcinoma is mainly driven by the stage of the tumor and by the overall liver function status. However, survival rates of patients with hepatocellular carcinoma are heterogeneous. In this study, we investigated whether circulating biomarkers might allow us to stratify the survival of patients with a new diagnosis of hepatocellular carcinoma. We observed that three biomarkers (namely AFP, PIVKA-II, and CYFRA 21-1) were independent predictors of overall survival. In addition, the combined use of these biomarkers allowed us to further stratify patients with hepatocellular carcinoma, according to their survival probability. This approach might help clinicians to tailor more personalized treatment strategies.

**Abstract:**

Keratin 19 (K19) is a cancer stem cell marker expressed by a subpopulation of hepatocellular carcinoma (HCC), associated with tumor aggressiveness. We evaluated the prognostic value of serum K19 fragment (CYFRA 21-1), in comparison or in combination with alpha-fetoprotein (AFP) and protein induced by vitamin-K absence or antagonist-II (PIVKA-II), in patients with HCC. A total of 160 patients (28F/132M; median age 62, range 44–86 years) with a new diagnosis of HCC and available serum samples collected at tumor diagnosis were analyzed retrospectively. Median overall survival (OS) after HCC diagnosis was 35.1, 95% CI 27.1–70.5 months. Multivariate Cox regression analysis showed that CYFRA 21-1 > 2.7 ng/mL (hazard ratio (HR) = 3.39, *p* < 0.001), AFP > 20 ng/mL (HR = 2.27, *p* = 0.007), and PIVKA-II > 200 mAU/mL (HR = 2.17, *p* = 0.020) were independent predictors of OS. The combination of biomarkers positivity allowed us to stratify patients with HCC into four risk categories associated with a progressively lower survival probability (log-rank test, *p* < 0.001). CYFRA 21-1 resulted an independent prognostic factor of patients with HCC and its combination with AFP and PIVKA-II might be useful to tailor personalized treatment strategies.

## 1. Introduction

Liver cancer is the sixth most common cancer in terms of incidence and the third in terms of mortality worldwide, with 841,000 new cases (4.7%; cumulative risk: 1.08) and 782,000 deaths (8.2%; cumulative risk: 0.98) per year [1]. Hepatocellular carcinoma (HCC) represents more than 90% of primary liver cancers, chronic viral hepatitis (B and C) being the most frequent underlying etiology [2]. Chronic inflammation that characterizes the natural history of chronic hepatitis leads to fibrosis progression, and overtime, it leads to cirrhosis, a condition at high risk for HCC development [3]. It is estimated that one-third of patients with cirrhosis will develop HCC during their lifetime [4].

The prognosis of HCC varies greatly according to tumor stage at the time of diagnosis and the overall liver function status. The European Association for the Study of the Liver (EASL) suggested the use of the Barcelona Clinic Liver Cancer (BCLC) staging system for HCC classification and treatment allocation [5], while no tissue and serum biomarkers were endorsed for predicting prognosis. Thus, the identification of novel surrogate biomarkers is crucial for further refinement of prognosis evaluation. Promising results were observed for alpha-fetoprotein (AFP) and protein induced by vitamin K absence or antagonist II (PIVKA-II), for monitoring treatment outcomes and predicting prognosis [6,7]. However, due to the heterogeneity of the studies, there is still no consensus regarding their role in clinical practice.

Keratin 19 (K19) is a hepatic progenitor cell marker associated with epithelial–mesenchymal transition; K19+ HCCs showed a more aggressive and metastatic phenotype compared to K19- tumors [8]. Recently, it was shown that the circulating levels of cytokeratin 19 fragment (CYFRA 21-1) are able to reflect tumor K19 expression [9]. In addition, functional experiments confirmed that CYFRA 21-1 levels were directly regulated by K19 function in HCC cells [9].

The aim of the present study was to evaluate the prognostic value of serum CYFRA 21-1, compared to AFP and PIVKA-II in patients with HCC, and to investigate whether the combined use of these biomarkers might allow an appropriate stratification of patients’ survival.

## 2. Results

The demographic, clinical, and biochemical characteristics of the patients enrolled are reported in Table 1. Median age was 62 (44–86) years and most patients were males (*n* = 132, 82.5%). All patients had cirrhosis and the main underlying etiology was viral (*n* = 118, 73.8%). Regarding tumor characteristics, 82 (51.2%) patients had a single nodular HCC, 42 (26.3%) had a multinodular HCC (2–3 lesions), and 36 (22.5%) had a multinodular HCC, with more than three lesions. Median tumor size was 3.1 (0.7–13.0) cm. According to the BCLC staging system, 18 (11.3%) patients had a very early HCC (BCLC stage 0), 77 (48.1%) patients had early HCC (BCLC stage A), 39 (24.4%) patients had an intermediate HCC (BCLC stage B), 23 (14.4%) patients had an advanced HCC (BCLC stage C), and 3 (1.9%) patients had an end-stage HCC (BCLC stage D).

As first-line therapy, 69 (43.1%) patients were treated with thermoablative percutaneous techniques—radiofrequency ablation (RFA) or microwave ablation (MWA) associated or not with percutaneous ethanol injection. Nine (5.6%) patients underwent surgery as curative treatment and 8 (5.0%) patients underwent orthotopic liver transplantation (OLT). Nine (5.6%) patients with early tumor, unfit for standard curative treatments, were treated with stereotactic ablative radiotherapy. Twenty-six (16.3%) patients received trans-arterial chemoembolization (TACE) while 5 (3.1%) patients underwent a combination approach (MWA or RFA associated with TACE) as a downstaging strategy for OLT or for further treatments. Trans-arterial radioembolization was allocated to 8 (5.0%) patients with intermediate HCC unsuitable for TACE. Twenty (12.5%) patients with advanced HCC were treated with systemic chemotherapy, based on sorafenib and 2 (9.4%) patients in the terminal stage received palliative support including management of pain, nutrition, and psychological support. Finally, 4 (2.5%) patients refused HCC treatment.

**Figure 1:**
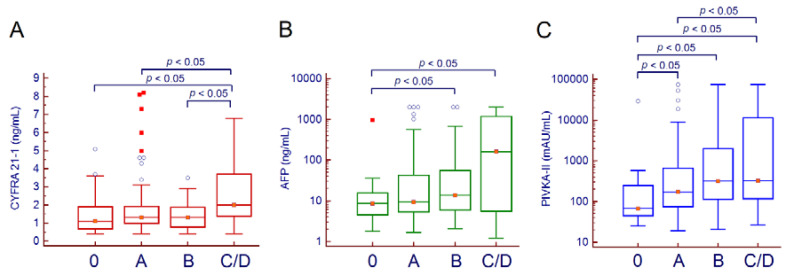
CYFRA 21-1 (**A**), AFP (**B**), and PIVKA-II (**C**) serum levels among the different BCLC stages. Due to the low number of patients with end-stage HCC (*n* = 3), patients with BCLC stage C and D were merged. Hollow circles indicate values that are larger than the upper quartile plus 1.5 times the interquartile range while red squares indicate values that are larger than the upper quartile plus 3 times the interquartile range. Abbreviations—alpha-fetoprotein (AFP), Barcelona Clinic Liver Cancer (BCLC), and protein induced by vitamin K absence or antagonist II (PIVKA-II).

At HCC diagnosis, median CYFRA 21-1, AFP, and PIVKA-II levels were 1.3, 95% confidence interval (CI) 1.2–1.5 ng/mL, 12.6, 95% CI 8.6–16.9 ng/mL, and 199, 95% CI 146–316 mAU/mL, respectively. Median serum CYFRA 21-1 was significantly higher in patients with HCC, compared to patients with cirrhosis and healthy subjects (Figure S1). Regarding demographics, no significant correlation was observed between age at HCC diagnosis and CYFRA 21-1 (*r_s_* = 0.003, 95% CI −0.152–0.158, *p* = 0.967), AFP (*r_s_* = −0.073, 95% CI −0.226–0.083, *p* = 0.360), and PIVKA-II (*r_s_* = 0.049, 95% CI −0.107–0.203, *p* = 0.537), and no differences in biomarker levels were observed according to gender (CYFRA 21-1: *p* = 0.526; AFP: *p* = 0.558; PIVKA-II: *p* = 0.077). Overall, CYFRA 21-1 and PIVKA-II serum levels varied significantly among the different BCLC stages (Kruskal-Wallis test, *p* = 0.008 and *p* = 0.004, respectively), while only a trend was observed for AFP (*p* = 0.051). Pairwise comparison of the BCLC stages for each biomarker is depicted in Figure 1.

CYFRA 21-1 serum levels were weakly correlated with PIVKA-II (*r_s_* = 0.163, 95% CI 0.008–0.311, *p* = 0.039) but not with AFP (*r_s_* = 0.044, 95% CI −0.112–0.198, *p* = 0.579); in addition, we observed a moderate positive correlation between CYFRA 21-1 and aspartate aminotransferase (AST) (*r_s_* = 0.321, 95% CI 0.173–0.455, *p* < 0.001), and a moderate negative correlation with serum albumin (*r_s_* = −0.454, 95% CI −0.572–−0.318, *p* < 0.001). AFP showed a weak correlation with alanine aminotransferase (ALT) (*r_s_* = 0.244, 95% CI 0.091–0.386, *p* = 0.001) and AST (*r_s_* = 0.295, 95% CI 0.145–0.432, *p* < 0.001), while no significant correlation was observed between PIVKA-II and the biochemical parameters (Figure S2).

### Prediction of Overall Survival

**Figure 2:**
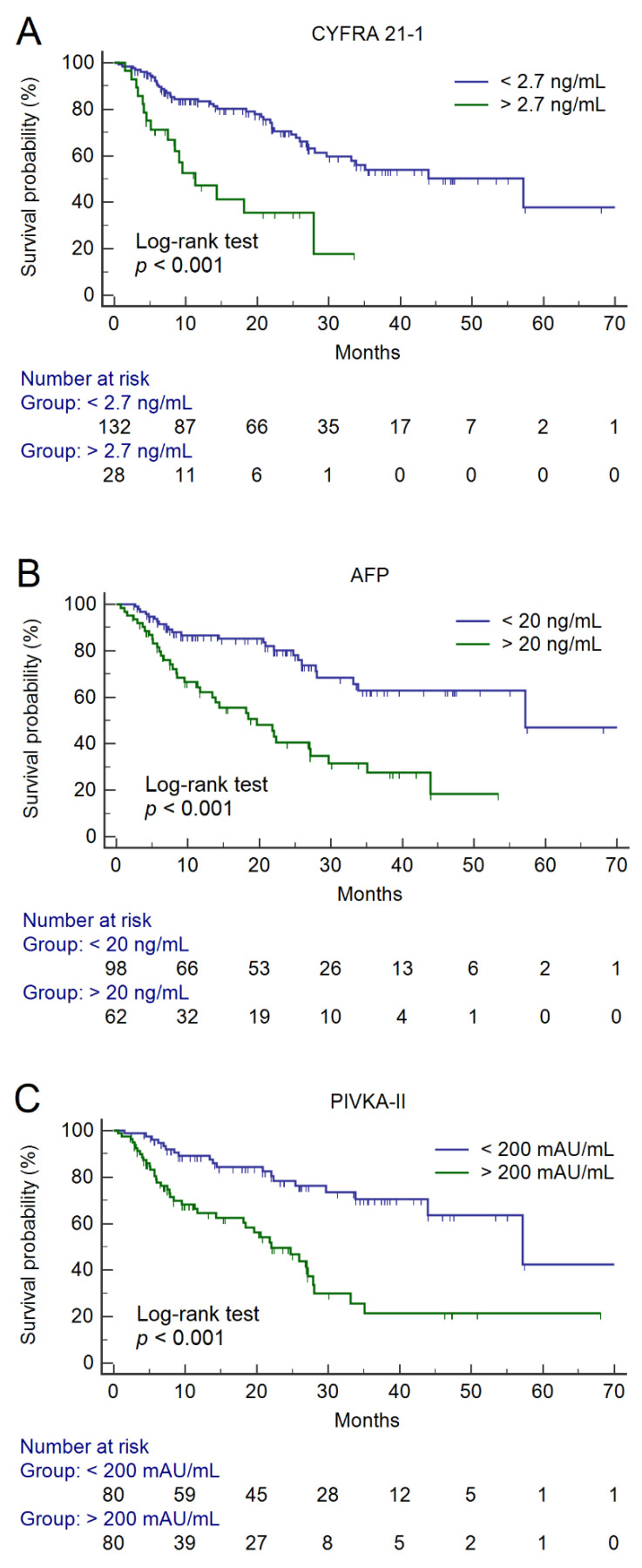
Survival curves according to baseline values of CYFRA 21-1 (**A**), AFP (**B**), and PIVKA-II (**C**). Abbreviations—alpha-fetoprotein (AFP) and protein induced by vitamin K absence or antagonist II (PIVKA-II).

The median overall survival (OS) after HCC diagnosis was 35.1, 95% CI 27.1–70.5 months. Sixty (37.5%) patients died during the follow-up period. At univariate analysis, we observed significantly different survival curves, according to baseline CYFRA 21-1 > 2.7 ng/mL (*p* < 0.001), AFP > 20 ng/mL (*p* < 0.001), and PIVKA-II > 200 mAU/mL (*p* < 0.001) (Figure 2).

The median OS was 57.2, 95% CI 29.7–70.5 months and 11.3, 95% CI 7.5–27.9 months, according to CYFRA 21-1 > 2.7 ng/mL; 57.2, 95% CI 33.8–70.5 months and 19.6, 95% CI 11.7–27.1 months according to AFP > 20 ng/mL; and 57.2, 95% CI 43.9–70.5 months and 22.0, 95% CI 18.1–27.9 months according to PIVKA-II > 200 mAU/mL, respectively. All three biomarkers were predictors of OS at univariate analysis. Through multivariate Cox proportional-hazard regression analysis, CYFRA 21-1 > 2.7 ng/mL (hazard ratio (HR) = 3.39, 95% CI 1.76–6.52, *p* < 0.001), AFP > 20 ng/mL (HR = 2.27, 95% CI 1.25–4.13, *p* = 0.007), and PIVKA-II > 200 mAU/mL (HR = 2.17, 95% CI 1.13–4.17, *p* = 0.020) results were significant and independent predictors of OS, irrespective of liver function (Child-Pugh Score), AST values, the BCLC stage of HCC, and the radiological response to treatment (Table 2).

**Figure 3:**
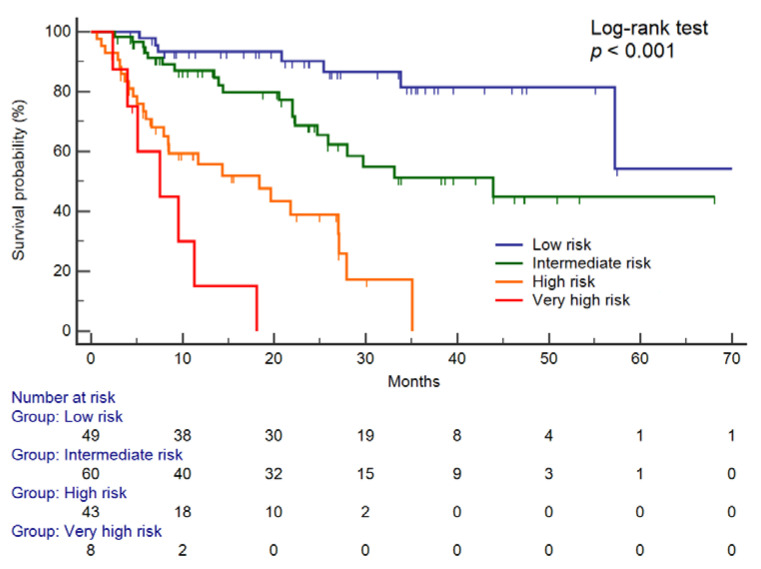
Survival curves according to risk categories. Based on the cut-off of 2.7 ng/mL for CYFRA 21-1, 20 ng/mL for AFP, and 200 mAU/mL for PIVKA-II, the low-risk category corresponded to no biomarker positivity, the intermediate risk category corresponded to the positivity to one biomarker, the high-risk category corresponded to the positivity to two biomarkers, and the very high-risk category corresponded to the positivity of all three biomarkers. Abbreviations—alpha-fetoprotein (AFP) and protein induced by vitamin K absence or antagonist II (PIVKA-II).

Four risk categories (low, intermediate, high, and very high) were created and based on the CYFRA 21-1, AFP, and PIVKA-II positivity, according to the selected cut-offs; the low-risk category corresponded to no biomarker positivity, the intermediate risk category corresponded to the positivity to one biomarker, the high-risk category corresponded to the positivity to two biomarkers and the very high-risk category corresponded to the positivity to all three biomarkers. Kaplan–Meier analysis showed a significant stepwise increase of the cumulative mortality, according to the four risk categories (Figure 3).

Median OS was 70.5, 95% CI 57.2–70.5 months for the low-risk group, 34.9, 95% CI 25.9–43.9 months for the intermediate risk group, 18.4, 95% CI 8.4–27.1 months for the high-risk group, and 7.5, 95% CI 4.0–11.3 months for the very high-risk group. Multivariate adjusted HRs for OS of the four risk categories are described in Table 3. As compared with the low-risk category, both the intermediate, high-risk, and very high-risk categories were significantly associated with a progressively higher likelihood of death.

## 3. Discussion

In the present study we showed that the measurement of serum CYFRA 21-1, AFP, and PIVKA-II at the time of HCC diagnosis allowed appropriate prediction of OS. Moreover, the combined use of these biomarkers permitted to further stratify the risk of death independently from the tumor stage and response to treatment, providing an unbiased prognostic tool that might be used clinically to refine the classification of patients with HCC and the allocation of treatment.

To date, CYFRA 21-1 is a well-established prognostic indicator of patients with non-small cell lung cancer [10], while research data on HCC are scanty. In the animal model, it was reported that CYFRA 21-1 levels increased in parallel with tumor progression and further rose when pulmonary metastases occurred [11]. In the clinical setting, the linifanib phase II trial showed that lower baseline concentrations CYFRA21-1, as well as PIVKA-II, were associated with improved OS in patients with unresectable or metastatic HCC [12]. Consistently, we observed that baseline serum CYFRA 21-1 levels differentiated patients with low (11.3 months) and high median OS (57.2 months); patients with CYFRA 21-1 values > 2.7 ng/mL showed a more than 3-fold risk of death during the follow-up period.

Similar to CYFRA 21-1, baseline AFP and PIVKA-II were also able to predict OS, regardless of the BCLC staging and the radiological response to treatment. In agreement with our findings, previous studies reported on the prognostic utility of baseline AFP and PIVKA-II, for patients with different tumor severities and treatment approaches [13,14,15]. In addition, Payancé et al. showed that the ratio between serum PIVKA-II levels within the first 3 months after initial HCC treatment and baseline, predicted OS in a French cohort of patients with HCC, independent of the BCLC score, tumor size and number, liver disease severity, and radiological tumor response [16].

Finally, by the combination of biomarkers positivity we were able to further stratify the survival rates into four distinct risk categories; this approach was recently demonstrated as beneficial for predicting the outcome for patients with HCC treated with local therapy [17,18]. Our results might be useful for the selection of the more appropriate treatment strategy particularly for patients who do not fulfill all criteria for the treatment allocation or for the identification of patients that might benefit from the treatment option recommended for the subsequent more advance tumor stage, rather than the first line treatment for that specific stage. Further studies including larger cohort of patients are needed to investigate this issue.

The present study might be limited by the size and heterogeneity of the population analyzed, making it difficult to perform any sub-analysis according to tumor stage or therapeutic approach. However, our analyses were adjusted for confounding factors such as BCLC stage and radiological response to treatment. Thus, we believe that the results are reasonably robust and highlight a potential clinical usefulness of the investigated biomarkers.

## 4. Materials and Methods

### 4.1. Patients

A total of 160 consecutive patients with cirrhosis and a new diagnosis of HCC from the outpatient clinic of the Unit of Gastroenterology of Città della Salute e della Scienza di Torino–Molinette Hospital, Turin, Italy were recruited in our retrospective study between November 2012 and January 2018. An additional group of 22 healthy subjects and 44 patients with cirrhosis, without HCC, was enrolled as control population.

The presence of cirrhosis was determined by liver elastography (FibroScan^®^, Echosens™, Paris, France) or by hepatic ultrasound features and endoscopic signs of portal hypertension [19,20]. The diagnosis of HCC was achieved by histological examination (*n* = 15, 9.4%) or by contrast-enhanced imaging methods (*n* = 145, 90.6%) showing the typical hallmark of HCC (i.e., the combination of hypervascularity in late arterial phase and washout on portal venous and/or delayed phases), following international guidelines [5,21]. BCLC staging system was adopted for patients’ classification. Therapy allocation followed BCLC staging system and, in some cases, a multidisciplinary approach was needed to choose the best therapeutic approach. Radiological response to treatment was assessed by modified Response Evaluation Criteria in Solid Tumors (mRECIST) [22].

All patients included in the study underwent venous blood sampling at the diagnosis of HCC; serum was collected in polypropylene 2 mL tubes labelled with the study participant identification code and stored at −80 °C, until analysis. Study procedures were compliant to the principles of the Declaration of Helsinki. All patients gave their written informed consent and the study was approved by the Institutional Ethics Committee (CEI-452).

### 4.2. Measurement of Serum CYFRA 21-1, AFP, and PIVKA-II

Serum levels of CYFRA 21-1, AFP, and PIVKA-II were determined on the fully automated chemiluminescent enzyme immunoassay (CLEIA) system Lumipulse^®^ G600 II (Fujirebio Inc., Tokyo, Japan) using Lumipulse^®^ G CYFRA, Lumipulse^®^ G AFP-N, and Lumipulse^®^ G PIVKA-II reaction cartridges, according to manufacturer’s instructions. CYFRA 21-1 and AFP concentrations were given in ng/mL, while PIVKA-II values were reported in mAU/mL [23]. Detection limit of CYFRA 21-1, AFP, and PIVKA-II assays were 0.5 ng/mL, 0.075 ng/mL, and 1.37 mAU/mL, respectively.

### 4.3. Statistical Analysis

The primary outcome was OS, which was determined by the number of months from diagnosis until death or last follow-up. Categorical variables were reported as absolute number and percentage (%), while continuous variables were reported as median and range or 95% CI. Data distribution was checked by the D’Agostino-Pearson test. Comparison of continuous variables between two independent groups was performed by Mann-Whitney test. Kruskal-Wallis was used to compare continuous variables among different groups (more than 2). If the Kruskal-Wallis test was positive (*p* < 0.05), a post-hoc Conover test for pairwise comparison of subgroups was performed. Comparison of categorical variables between independent groups was performed by chi-squared (χ^2^) test. Correlation between variables was performed by non-parametric Spearman correlation test (*r_s_*). The survival curves were estimated by the Kaplan–Meier method with the log-rank test. For survival analysis, we adopted the widely used cut-off values of 20 ng/mL for AFP and 200 mAU/mL for PIVKA-II [24,25,26,27]; for CYFRA 21-1 we applied the cut-off value of 2.7 ng/mL, which was previously proposed by Kawai and colleagues [9]. Multivariate Cox proportional-hazard regression analysis was performed to evaluate whether variables associated to OS at univariate analysis were independently associated with patient survival. All statistical analyses were performed by using MedCalc^®^ v.18.9.1 (MedCalc Software Ltd., Ostend, Belgium) and a *p*-value smaller than 0.05 was considered to be statistically significant.

## 5. Conclusions

CYFRA 21-1 resulted a significant prognostic factor that is able to predict the survival of patients with HCC, independent of the tumor stage and response to treatment. The combined use of CYFRA21-1, AFP, and PIVKA-II might be useful for the refinement of BCLC classification and might help clinicians to tailor personalized treatment strategies.

## Figures and Tables

**Table 1:** Characteristics of the 160 patients with hepatocellular carcinoma (HCC).

Characteristics	Patients (*n* = 160)
Age, years (median and range)	62 (44–86)
Gender, M/F	132/28
BMI, Kg/m^2^ (median, 95% CI)	25.9 (25.3–26.5)
Smoking (*n*, %)	
Current	52 (32.5%)
Ex	60 (37.5%)
No	48 (30.0%)
Alcohol consumption *(*n*, %)	25 (15.6%)
Etiology (*n*, %)	
HCV	98 (61.3%)
HBV	20 (12.5%)
Non-viral	42 (26.2%)
Child-Pugh Score (*n*, %)	
A	119 (74.4%)
B	37 (23.1%)
C	4 (2.5%)
Esophageal Varices (*n*, %)	
No	99 (61.9%)
F1	24 (15.0%)
F2	33 (20.6%)
F3	4 (2.5%)
Ascites (*n*, %)	37 (23.1%)
ALT, IU/L (median, 95% CI)	43 (37–49)
AST, IU/L (median, 95% CI)	54 (45–59)
Haemoglobin, g/dL (median, 95% CI)	13.7 (13.2–13.9)
Platelet count, ×10^9^/L (median, 95% CI)	115 (106–127)
Albumin, g/dL (median, 95% CI)	4.0 (3.8–4.1)
INR (median, 95% CI)	1.15 (1.12–1.16)
Total Bilirubin, mg/dL (median 95% CI)	1.0 (0.9–1.1)
Creatinin, mg/dL (median 95% CI)	0.82 (0.77–0.87)
BCLC staging (*n*, %)	
0	18 (11.3%)
A	77 (48.1%)
B	39 (24.4%)
C	23 (14.4%)
D	3 (1.9%)

* >20 g/day in females and >40 g/day in males. Abbreviations—alanine aminotransferase (ALT), aspartate aminotransferase (AST), Barcelona Clinic Liver Cancer (BCLC) staging, body mass index (BMI), confidence interval (CI), female (F), hepatitis B virus (HBV), hepatocellular carcinoma (HCC), hepatitis C virus (HCV), international normalized ratio (INR), and male (M).

**Table 2:** Univariate and multivariate Cox proportional-hazard regression analysis of the predictors of overall survival.

Variable	Univariate		Multivariate	
	HR, 95% CI	*p* Value	HR, 95% CI	*p* Value
Age, years	1.01, 0.99–1.05	0.246	//	//
Gender, M	1.52, 0.75–3.10	0.248	//	//
BMI, Kg/m^2^	1.06, 0.99–1.14	0.092	//	//
Current smoking	1.38, 0.80–2.41	0.250	//	//
Alcohol consumption *	1.03, 0.55–1.95	0.923	//	//
Etiology, non-viral	0.98, 0.56–1.74	0.956	//	//
Child-Pugh Score, A	0.48, 0.27–0.84	0.010	0.96, 0.56–1.64	0.888
Esophageal Varices, absent	0.62, 0.36–1.06	0.081	//	//
ALT, IU/L	1.00, 0.99–1.01	0.594	//	//
AST, IU/L	1.01, 1.00–1.01	0.012	1.00, 0.99–1.01	0.247
Haemoglobin, g/dL	0.98, 0.79–1.01	0.061	//	//
Platelet count, ×10^9^/L	0.99, 0.99–1.00	0.600	//	//
Creatinin, mg/dL	1.13, 0.71–1.80	0.598	//	//
BCLC staging	2.10, 1.57–2.81	<0.001	1.60, 1.16–2.22	0.004
Radiological response	0.11, 0.04–0.26	<0.001	0.16, 0.06–0.45	0.001
CYFRA 21-1 > 2.7 ng/mL	3.27, 1.81–5.93	<0.001	3.39, 1.76–6.52	<0.001
AFP > 20 ng/mL	3.17, 1.87–5.38	<0.001	2.27, 1.25–4.13	0.007
PIVKA-II > 200 mAU/mL	3.40, 1.95–5.91	<0.001	2.17, 1.13–4.17	0.020

* >20 g/day in females and >40 g/day in males. Abbreviations—alpha-fetoprotein (AFP), alanine aminotransferase (ALT), aspartate aminotransferase (AST), Barcelona Clinic Liver Cancer (BCLC), Body Mass Index (BMI), confidence interval (CI), hazard ratio (HR), male (M), and protein induced by vitamin K absence or antagonist II (PIVKA-II), variable not included in the multivariate analysis (//).

**Table 3:** Cumulative probability of death and multivariate adjusted hazard ratios by risk category.

Risk Category	Events	Cumulative	HR, 95% CI *	*p* Value
Low	8/49	16.3%	1.00	reference
Intermediate	20/60	33.3%	3.92, 1.54–9.99	0.004
High	25/43	58.1%	7.69, 3.09–19.12	<0.001
Very high	7/8	87.5%	16.27, 5.14–51.50	<0.001

* Adjusted by BCLC stage and radiological response to treatment. Abbreviations—Barcelona Clinic Liver Cancer (BCLC), confidence interval (CI), and hazard ratio (HR).

## Supplementary Materials

The following are available online at https://www.mdpi.com/2072-6694/12/10/2776/s1, Figure S1: Serum values of CYFRA 21-1 in healthy subjects, patients with cirrhosis without HCC and patients with HCC, Figure S2: Correlation between HCC biomarkers and biochemical parameters.

## References

[1] The Global Cancer Observatory-IARC. https://gco.iarc.fr/today.

[2] Akinyemiju T., Abera S., Ahmed M., Alam N., Alemayohu M.A., Allen C., Al-Raddadi R., Alvis-Guzman N., Amoako Y., Artaman A. (2017). The Burden of Primary Liver Cancer and Underlying Etiologies From 1990 to 2015 at the Global, Regional, and National Level: Results from the Global Burden of Disease Study 2015. JAMA Oncol..

[3] Caviglia G.P., Rosso C., Fagoonee S., Saracco G.M., Pellicano R. (2017). Liver fibrosis: The 2017 state of art. Panminerva Med..

[4] Sangiovanni A., Prati G.M., Fasani P., Ronchi G., Romeo R., Manini M., Del Ninno E., Morabito A., Colombo M. (2006). The natural history of compensated cirrhosis due to hepatitis C virus: A 17-year cohort study of 214 patients. Hepatology.

[5] European Association for the Study of the Liver (2018). EASL Clinical Practice Guidelines: Management of hepatocellular carcinoma. J. Hepatol..

[6] Mazzaferro V., Sposito C., Zhou J., Pinna A.D., De Carlis L., Fan J., Cescon M., Di Sandro S., Yi-Feng H., Lauterio A. (2018). Metroticket 2.0 model for analysis of competing risks of death following liver transplantation for hepatocellular carcinoma. Gastroenterology.

[7] Park H., Kim S.U., Park J.Y., Kim D.Y., Ahn S.H., Chon C.Y., Han K.H., Seong J. (2014). Clinical usefulness of double biomarkers AFP and PIVKA-II for subdividing prognostic groups in locally advanced hepatocellular carcinoma. Liver Int..

[8] Kawai T., Yasuchika K., Ishii T., Katayama H., Yoshitoshi E.Y., Ogiso S., Kita S., Yasuda K., Fukumitsu K., Mizumoto M. (2015). Keratin 19, a Cancer Stem Cell Marker in Human Hepatocellular Carcinoma. Clin. Cancer Res..

[9] Kawai T., Yasuchika K., Ishii T., Katayama H., Yoshitoshi E.Y., Ogiso S., Minami T., Miyauchi Y., Kojima H., Yamaoka R. (2017). Identification of keratin 19-positive cancer stem cells associating human hepatocellular carcinoma using CYFRA 21-1. Cancer Med..

[10] Xu Y., Xu L., Qiu M., Wang J., Zhou Q., Xu L., Wang J., Yin R. (2015). Prognostic value of serum cytokeratin 19 fragments (Cyfra 21-1) in patients with non-small cell lung cancer. Sci. Rep..

[11] Ding S.J., Li Y., Tan Y.X., Jiang M.R., Tian B., Liu Y.K., Shao X.X., Ye S.L., Wu J.R., Zeng R. (2004). From proteomic analysis to clinical significance: Overexpression of cytokeratin 19 correlates with hepatocellular carcinoma metastasis. Mol. Cell. Proteom..

[12] Toh H.C., Chen P.J., Carr B.I., Knox J.J., Gill S., Ansell P., McKeegan E.M., Dowell B., Pedersen M., Qin Q. (2013). Phase 2 trial of linifanib (ABT-869) in patients with unresectable or metastatic hepatocellular carcinoma. Cancer.

[13] Galle P.R., Foerster F., Kudo M., Chan S.L., Llovet J.M., Qin S., Schelman W.R., Chintharlapalli S., Abada P.B., Sherman M. (2019). Biology and significance of alpha-fetoprotein in hepatocellular carcinoma. Liver Int..

[14] Yang M., Zhang X., Liu J. (2019). Prognostic value of des-γ-carboxy prothrombin in patients with hepatocellular carcinoma treated with transarterial chemotherapy: A systematic review and meta-analysis. PLoS ONE.

[15] Bai D.S., Zhang C., Chen P., Jin S.J., Jiang G.Q. (2017). The prognostic correlation of AFP level at diagnosis with pathological grade, progression, and survival of patients with hepatocellular carcinoma. Sci. Rep..

[16] Payancé A., Dioguardi Burgio M., Peoc’h K., Achahboun M., Albuquerque M., Devictor J., Chor H., Manceau H., Soubrane O., Durand F. (2019). Biological response under treatment and prognostic value of protein induced by vitamin K absence or antagonist-II in a French cohort of patients with hepatocellular carcinoma. Eur. J. Gastroenterol. Hepatol..

[17] Ryu T., Takami Y., Wada Y., Tateishi M., Matsushima H., Mikagi K., Saitsu H. (2017). Double- and Triple-Positive Tumor Markers Predict Early Recurrence and Poor Survival in Patients with Hepatocellular Carcinoma within the Milan Criteria and Child-Pugh Class, A.J. Gastrointest. Surg..

[18] Nitta H., Nakagawa S., Kaida T., Arima K., Higashi T., Taki K., Okabe H., Hayashi H., Hashimoto D., Chikamoto A. (2017). Pre-treatment double- or triple-positive tumor markers are predictive of a poor outcome for patients undergoing radiofrequency ablation for hepatocellular carcinoma. Surg. Today.

[19] Caviglia G.P., Touscoz G.A., Smedile A., Pellicano R. (2014). Noninvasive assessment of liver fibrosis: Key messages for clinicians. Pol. Arch. Med. Wewn..

[20] Gaia S., Campion D., Evangelista A., Spandre M., Cosso L., Brunello F., Ciccone G., Bugianesi E., Rizzetto M. (2015). Non-invasive score system for fibrosis in chronic hepatitis: Proposal for a model based on biochemical, FibroScan and ultrasound data. Liver Int..

[21] Matsui O., Kobayashi S., Sanada J., Kouda W., Ryu Y., Kozaka K., Kitao A., Nakamura K., Gabata T. (2011). Hepatocellular nodules in liver cirrhosis: Hemodynamic evaluation (angiography-assisted CT) with special reference to multi-step hepatocarcinogenesis. Abdom. Imaging.

[22] Lencioni R., Llovet J.M. (2010). Modified RECIST (mRECIST) assessment for hepatocellular carcinoma. Semin. Liver Dis..

[23] Caviglia G.P., Abate M.L., Gaia S., Petrini E., Bosco C., Olivero A., Rosso C., Ciancio A., Pellicano R., Saracco G.M. (2017). Risk of hepatocellular carcinoma in HBV cirrhotic patients assessed by the combination of miR-122, AFP and PIVKA-II. Panminerva Med..

[24] Silva J.P., Gorman R.A., Berger N.G., Tsai S., Christians K.K., Clarke C.N., Mogal H., Gamblin T.C. (2017). The prognostic utility of baseline alpha-fetoprotein for hepatocellular carcinoma patients. J. Surg. Oncol..

[25] Gurakar A., Ma M., Garonzik-Wang J., Kim A., Anders R.A., Oshima K., Georgiades A., Gurakar M., Ottmann S., Cameron A.M. (2018). Clinicopathological distinction of low-AFP-secreting vs. High-AFP-secreting hepatocellular carcinoma. Ann. Hepatol..

[26] Kim J.M., Hyuck C., Kwon D., Joh J.W., Lee J.H., Paik S.W., Park C.K. (2013). Protein induced by vitamin K antagonist-II (PIVKA-II) is a reliable prognostic factor in small hepatocellular carcinoma. World J. Surg..

[27] Yu R., Tan Z., Xiang X., Dan Y., Deng G. (2017). Effectiveness of PIVKA-II in the detection of hepatocellular carcinoma based on real-world clinical data. BMC Cancer.

