# Supplementary material for: Prognostic Role of Serum Cytokeratin-19 Fragment (CYFRA 21-1) in Patients with Hepatocellular Carcinoma
Source: Cancers (Basel). 2020 Sep 28;12(10):2776. doi: 10.3390/cancers12102776 (PMC7601489; doi:10.3390/cancers12102776)
Supplement: Supplementary file 1 [file cancers-12-02776-s001.pdf]

Prognostic Role of Serum Cytokeratin-19 Fragment (CYFRA 21-1) in Patients with Hepatocellular Carcinoma

Gian Paolo Caviglia, Michela Ciruolo, Antonella Olivero, Patrizia Carucci, Emanuela Rolle, Chiara Rosso, Maria Lorena Abate, Alessandra Risso, Davide Giuseppe Ribaldone, Francesco Tandoi, Giorgio Maria Saracco, Elisabetta Bugianesi and Silvia Gaia

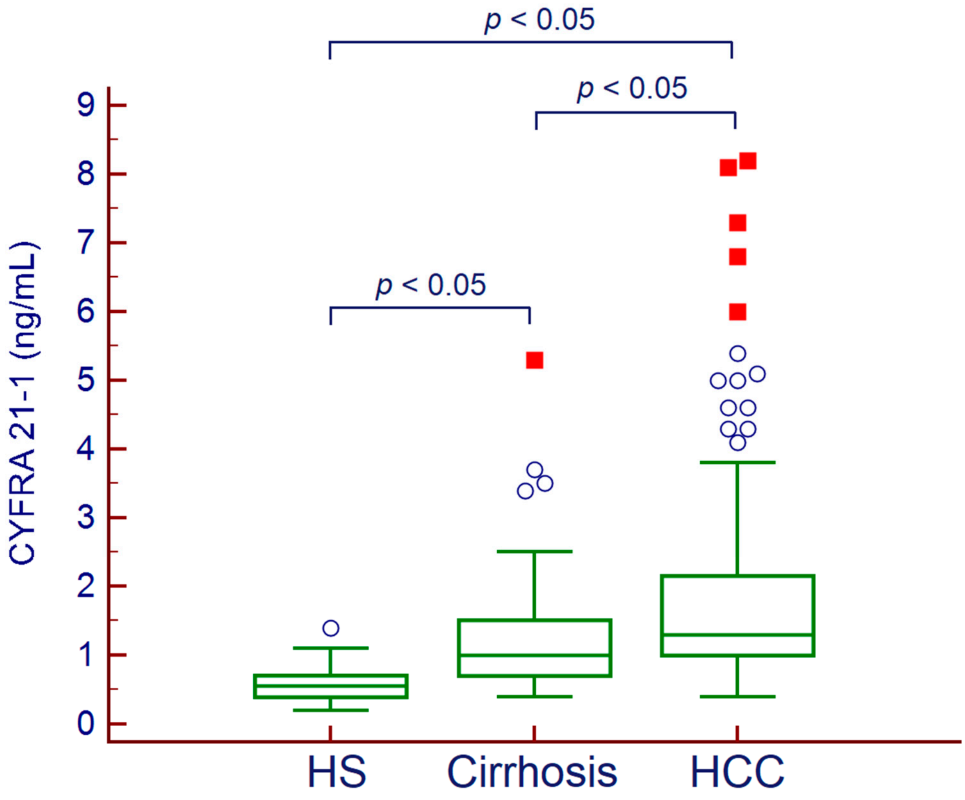

Figure S1. Serum values of CYFRA 21-1 in healthy subjects, patients with cirrhosis without HCC and patients with HCC. CYFRA 21-1 was measured in 22 HS (median age 58, range 43–63 years, 15 males and 7 females) in 44 patients with cirrhosis without HCC (median age 56, range 44–64 years, 31 males and 13 females) and 160 patients with HCC (median age 62, range 44–76 years, 132 males and 28 females). Serum CYFRA 21-1 progressively increased from HS (0.6, 95% CI 0.5–0.7 ng/mL) to patients with cirrhosis without HCC (1.0, 95% CI 0.7–1.1 ng/mL), and further raised in patients with HCC (1.3, 95% CI 1.2–1.5 ng/mL) (Kruskal-Wallis, $p < 0.001$). There were no significant differences between patients with cirrhosis without HCC and those with HCC regarding Child-Pugh Score (37 A, 7B vs. 119 A, 37 B, 4 C, respectively; χ^2 test, $p = 0.307$) and liver disease etiology (25 HCV, 10 HBV, 9 non-viral vs. 98 HCV, 20 HBV, 42 non-viral, respectively; χ^2 test, $p = 0.220$). Hollow circles indicate values that are larger than the upper quartile plus 1.5 times the interquartile range while red squares indicate values that are larger than the upper quartile plus 3 times the interquartile range. Abbreviations: hepatocellular carcinoma (HCC), healthy subjects (HS).

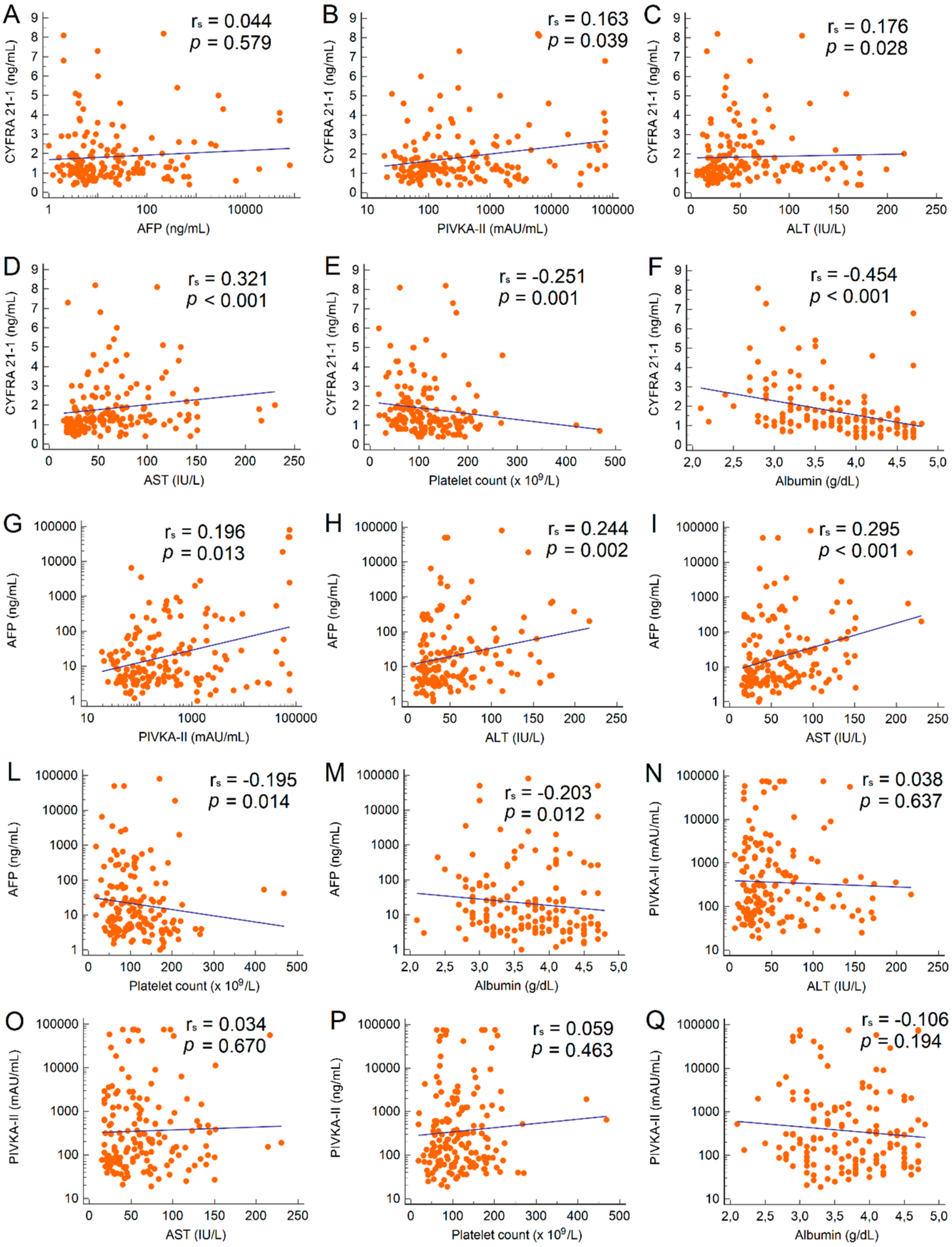

Figure S2. Correlation between HCC biomarkers and biochemical parameters. Abbreviations: alpha fetoprotein (AFP), alanine aminotransferase (ALT), aspartate aminotransferase (AST), international normalized ratio (INR), protein induced by vitamin K absence or antagonist II (PIVKA-II).

© 2020 by the authors. Licensee MDPI, Basel, Switzerland. This article is an open access article distributed under the terms and conditions of the Creative Commons Attribution (CC BY) license (http://creativecommons.org/licenses/by/4.0/).
